# Radiopharmaceuticals: navigating the frontier of precision medicine and therapeutic innovation

**DOI:** 10.1186/s40001-023-01627-0

**Published:** 2024-01-05

**Authors:** Shivang Dhoundiyal, Shriyansh Srivastava, Sachin Kumar, Gaaminepreet Singh, Sumel Ashique, Radheshyam Pal, Neeraj Mishra, Farzad Taghizadeh-Hesary

**Affiliations:** 1 Department of Pharmacy, School of Medical and Allied Sciences, Galgotias University, Greater Noida, 203201 India; 2 Department of Pharmacology, Delhi Pharmaceutical Sciences and Research University (DPSRU), Sector 3 Pushp Vihar, New Delhi, 110017 India; 3 Department of Physiology and Biophysics, Case Western Reserve University (CWRU), Cleveland, OH USA; 4 Department of Pharmaceutical Sciences, Bengal College of Pharmaceutical Sciences & Research, Durgapur, 713212 West Bengal India; 5 Department of Pharmacology, Pandaveswar School of Pharmacy, Pandaveswar, 713346 West Bengal India; 6 Amity Institute of Pharmacy, Amity University Madhya Pradesh, Gwalior, 474005 MP India; 7 ENT and Head and Neck Research Center and Department, The Five Senses Health Institute, School of Medicine, Iran University of Medical Sciences, Tehran, Iran; 8 Department of Clinical Oncology, Iran University of Medical Sciences, Tehran, Iran

**Keywords:** Radiopharmaceuticals, Positron emission tomography, Single-photon emission computed tomography, Drug delivery, Cancer

## Abstract

This review article explores the dynamic field of radiopharmaceuticals, where innovative developments arise from combining radioisotopes and pharmaceuticals, opening up exciting therapeutic possibilities. The in-depth exploration covers targeted drug delivery, delving into passive targeting through enhanced permeability and retention, as well as active targeting using ligand-receptor strategies. The article also discusses stimulus-responsive release systems, which orchestrate controlled release, enhancing precision and therapeutic effectiveness. A significant focus is placed on the crucial role of radiopharmaceuticals in medical imaging and theranostics, highlighting their contribution to diagnostic accuracy and image-guided curative interventions. The review emphasizes safety considerations and strategies for mitigating side effects, providing valuable insights into addressing challenges and achieving precise drug delivery. Looking ahead, the article discusses nanoparticle formulations as cutting-edge innovations in next-generation radiopharmaceuticals, showcasing their potential applications. Real-world examples are presented through case studies, including the use of radiolabelled antibodies for solid tumors, peptide receptor radionuclide therapy for neuroendocrine tumors, and the intricate management of bone metastases. The concluding perspective envisions the future trajectory of radiopharmaceuticals, anticipating a harmonious integration of precision medicine and artificial intelligence. This vision foresees an era where therapeutic precision aligns seamlessly with scientific advancements, ushering in a new epoch marked by the fusion of therapeutic resonance and visionary progress.

**Graphical Abstract:**

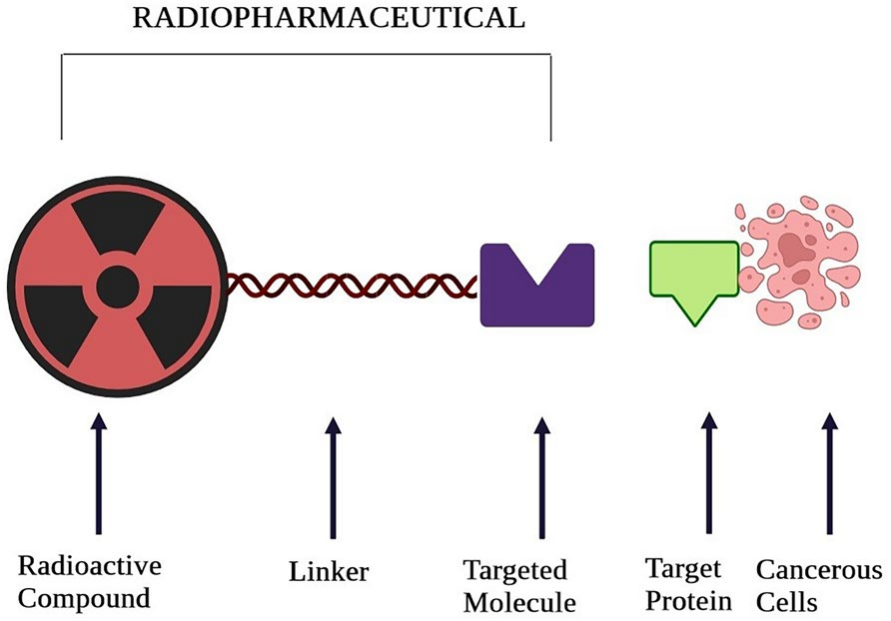

## Introduction

Radiopharmaceuticals serve as a crucial intersection of medicine, chemistry, and nuclear physics, playing a vital role in diagnostic imaging and targeted therapies. These compounds, meticulously engineered through the fusion of biologically active molecules and radioisotopes, offer non-invasive insights into molecular processes at cellular levels [1]. Diagnostic procedures like PET, SPECT, and scintigraphy utilize radiopharmaceuticals for early disease detection, leading to improved interventions [2]. Beyond diagnostics, radiopharmaceuticals are integral to targeted radionuclide therapy, a precise method of delivering radioactive payloads to diseased tissues. This approach enhances treatment efficacy while minimizing damage to healthy tissues, ultimately improving patient outcomes [3]. The integration of radiopharmaceuticals into therapeutic protocols reveals a captivating relationship of pharmacokinetics and biodistribution, governing their intricate journey within the human body. Understanding their absorption, distribution, metabolism, and excretion is crucial for shaping clinical protocols. Many studies have not only explored the mechanisms dictating radiopharmaceutical behavior but also unveiled the dynamic interplay between chemical composition, physiological context, and molecular recognition. Moreover, recent advancements in radiopharmaceutical research have introduced innovative strategies for enhancing their specificity and therapeutic effectiveness [4]. The development of novel targeting agents, improved radionuclide selection, and innovative delivery systems contribute to the evolving landscape of radiopharmaceutical applications [5]. In essence, the pharmacokinetics and biodistribution of radiopharmaceuticals provide a rich narrative on the canvas of human physiology, guiding practitioners and researchers to unlock the full potential of these marvels in healthcare. The continual refinement of these compounds and their applications promises even greater strides in the realms of diagnosis and targeted therapy, offering new avenues for improving patient care and outcomes.

### Fusion of radioisotopes and pharmaceuticals

The combination of radioisotopes and medicines is like a key part of modern medicine, bringing together science and innovation to transform how we diagnose, treat, and understand diseases [6]. These special pairings, known as radiopharmaceuticals, are created using a mix of chemistry, nuclear science, and biotechnology. They have this amazing ability to go through our bodies, uncovering hidden details about how things work on a molecular level. The use of radioisotopes, kind of like invisible markers, lets these medicines go beyond traditional drugs [7]. This opens up a new era in personalized medicine, allowing doctors to customize treatments based on each patient’s unique characteristics, making treatments more effective and reducing side effects. The combination of radioisotopes and medicines has also led to cool imaging techniques that show what’s happening inside our bodies. This helps with early detection, accurate understanding of diseases, and keeping track of how they change over time [8]. In simple terms, the blend of radioisotopes and medicines is like a new era of understanding and healing, where the coming together of these scientific fields creates a powerful force for the betterment of humanity. In essence, this collaboration between radioisotopes and medicines is more than a scientific breakthrough; it's a powerful force for the well-being of humanity. It is a journey into understanding, healing, and unlocking the secrets of our bodies for a healthier tomorrow.

### Radiolabelling: transforming compounds into radiopharmaceuticals

Radiolabelling is a radical approach in the world of radiopharmaceuticals, bringing a new level of precision to how we diagnose and treat using nuclear medicine. Essentially, it is about adding a tiny bit of radioactivity to a special compound, making it a powerful tool to target and illuminate specific processes inside the body [9]. This process involves a mix of chemistry, biology, and physics to carefully attach the radioactive tag without messing up the compound's natural activity. These radiolabelled compounds, now armed with radioactive ability, allow doctors and researchers to see, measure, and adjust what is happening in the body at the molecular level [10]. For example, owing to recent advancements, we now have new tracers for neuroimaging that help map brain functions and detect neurological disorders early on. As we keep advancing in radiolabelling, we are getting better at making radiopharmaceuticals. This opens up exciting possibilities for personalized medicine, early disease detection, and more effective therapies. But it is a meticulous process at the tiny molecular scale, where choices like which radioactive material to use, the chemistry of the linking agent, and how to connect things, all need careful planning to ensure stability and effectiveness. Beyond the lab, radiolabelling is making innovative contributions in real-world medical applications. For instance, we are now using radiolabelled peptides in targeted cancer therapy [11]. These special compounds travel through the bloodstream, giving us a closer look at cells and revealing the secrets of diseases under advanced imaging techniques. But the story does not stop there; radiolabelling is not just about diagnosis. In treating diseases, especially cancer, it becomes a form of art. Guided by the compound's affinity for specific targets, radiation therapy becomes a precise and effective way to triumph over diseases [12]. In the big picture of modern medicine, radiolabelling is like a stroke of genius, blending science and compassion. It is lighting up the way for better diagnostics, personalized treatments, and a deeper understanding of the intricate dance of life. The use of radiolabelling in molecular imaging and targeted therapeutics is a perfect example of how science and medicine working together can make a big impact on patient care and our understanding of complex biological processes [8].

## Targeted drug delivery strategies

**Fig. 1:**
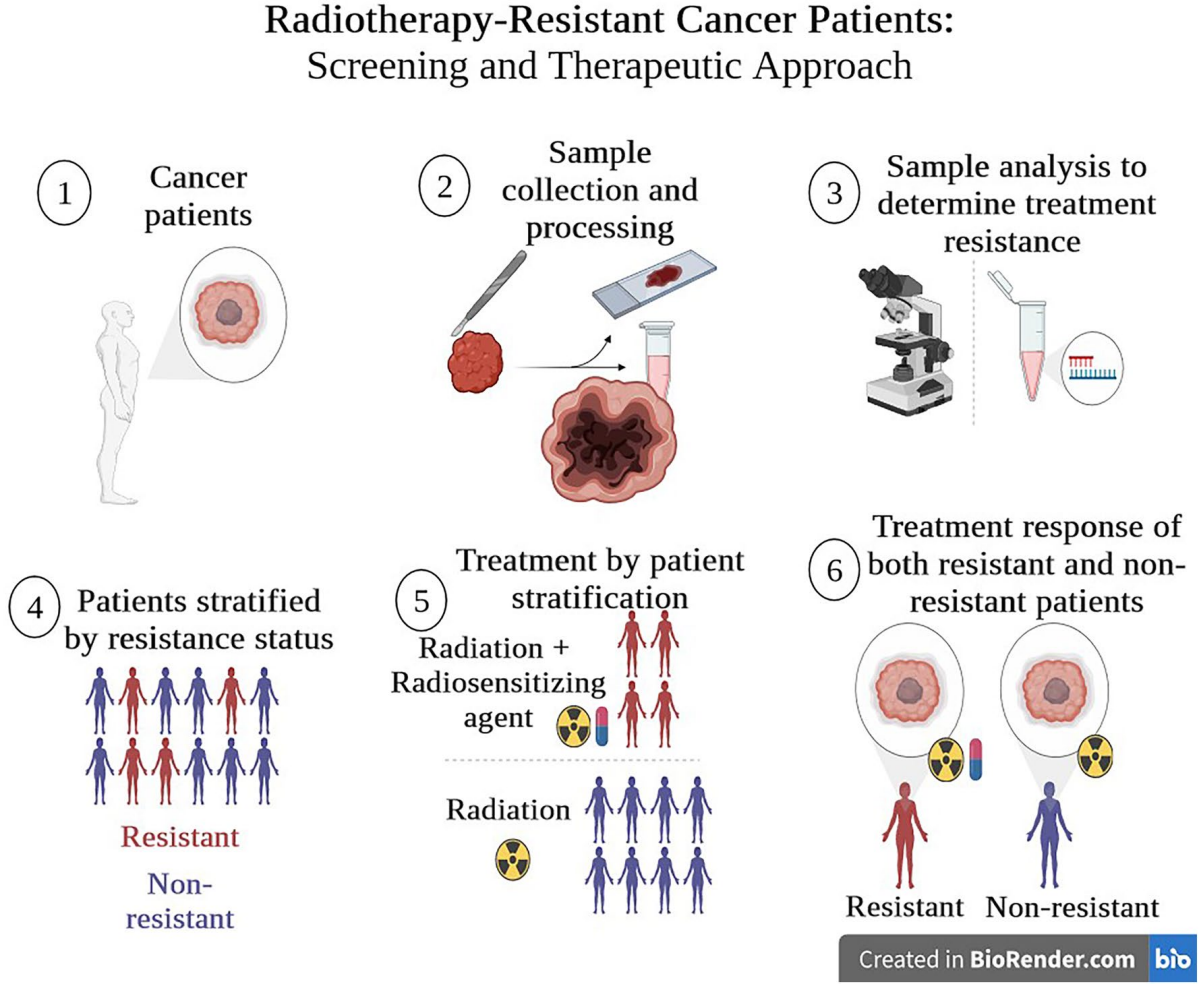
Radiotherapy response on resistant and non-resistant cancer patients

In the world of radiopharmaceuticals, targeted drug delivery is a carefully crafted interplay between medicine and tiny molecular engineering. However, it is changing how we treat diseases in a big way. These special medicines have a natural attraction to specific places in our bodies that need help. It is like guiding these medicines with incredible precision to exactly where they are needed the most [13]. This collaboration between advanced labeling techniques and drug design means these medicines not only hit the right spots but also avoid causing trouble in other areas of our bodies [14]. Whether it is dealing with cancer or conditions in our brain, this targeted delivery is making treatments more effective while keeping patients feeling better. It is like a teamwork of different scientific fields coming together to create something amazing [15]. This approach to targeted radiopharmaceuticals is like reaching the highest point of scientific progress, paving the way for personalized medicine, as depicted in Fig. 1 the role of radiotherapy in cancer patients. It is a new era where treatments are super precise, like a musical symphony of molecular specificity.

### Passive targeting: enhanced permeability and retention

Passive targeting is a sophisticated method in the field of radiopharmaceuticals, and it becomes particularly intriguing when we delve into the concept of Enhanced Permeability and Retention (EPR) [16]. Essentially, this strategy leverages the unique features of tumor environments, specifically their distinct blood vessels and compromised drainage systems. Carefully crafted radiopharmaceuticals, equipped with both therapeutic and diagnostic elements, navigate the complex landscape of the body, homing in precisely on tumor sites with remarkable precision [17]. As these meticulously designed constructs circulate through the body, they discreetly penetrate cancerous regions, utilizing the EPR effect to linger, accumulate, and gradually release their potent cargo over time. This innovative strategy achieves a dual victory: it minimizes harm to healthy tissues while maximizing the therapeutic impact at the core of the disease. With passive targeting and EPR guiding the way, radiopharmaceuticals are charting an unprecedented path toward refined treatment approaches, driven by scientific expertise and the intricate dance of biology. This offers a glimpse into a future where precision and compassion come together to enhance patient outcomes [18].

Recent advancements in passive targeting have been notable in optimizing the design of radiolabelled nanocarriers. Nanocarriers, including liposomes, micelles, polymers, dendrimers, and various nanoparticles, such as those made of gold, iron oxide, silica, or carbon, are being carefully designed to enhance their circulation time, biodistribution, and tumor penetration [19]. Modifications with ligands, coatings, or stimuli-responsive materials further improve their passive targeting performance. Additionally, passive targeting is advancing through the exploration of the EPR effect in combination with other targeting strategies. This involves integrating active targeting strategies, where ligands bind to receptors or antigens expressed on target cells. Moreover, triggered targeting, relying on external or internal stimuli like temperature, pH, light, ultrasound, or enzymes, is being investigated to enhance the selectivity and affinity of drug carriers [20]. A critical aspect of these advancements is the evaluation of the EPR effect and its variability in different tumors and patients. Recognizing that the EPR effect is not universal and can vary significantly, researchers are developing methods to measure and predict its occurrence in vivo. This involves utilizing imaging techniques, biomarkers, or mathematical models to personalize treatment regimens by optimizing the dose and timing of radiopharmaceutical administration, ultimately improving treatment outcomes.

### Active targeting: ligand-receptor strategies

Active targeting is a strategic approach in which a ligand, such as an antibody, peptide, or small molecule, is attached to a radioactive molecule, like a radioisotope. This method is designed to deliver radiation therapy directly to cancer cells that express specific receptors, thereby enhancing therapeutic efficacy and minimizing the toxicity of radiopharmaceuticals. Several successful examples of radiopharmaceuticals utilizing active targeting include Lutetium-177 dotatate (Lutathera), which targets the somatostatin receptor overexpressed in neuroendocrine tumors. Approved by the FDA in 2018 for treating gastroenteropancreatic neuroendocrine tumors, a phase III clinical trial demonstrated improved progression-free survival and overall response rates compared to standard therapy [21]. Another example is Radium-223 dichloride (Xofigo), which targets the bone matrix, preferentially accumulating in bone metastases. FDA-approved in 2013 for metastatic castration-resistant prostate cancer, a phase III clinical trial revealed enhanced overall survival and delayed symptomatic skeletal events compared to placebo [22]. Iodine-131 tositumomab (Bexxar) targets the CD20 antigen expressed on B cells and B-cell lymphomas. Approved by the FDA in 2003 for relapsed or refractory non-Hodgkin lymphoma, a phase III clinical trial demonstrated improved overall response rates and duration of response compared to rituximab, another CD20-targeting antibody [23]. These examples showcase the effectiveness of active targeting in tailoring radiation therapy to specific cancer cells, marking a significant advancement in precision medicine.

### Stimulus-responsive release systems

Stimulus-responsive release systems represent a cutting-edge approach in drug delivery, allowing the controlled release of active pharmaceutical ingredients (API) in response to specific conditions or stimuli such as pH, temperature, redox, light, or magnetic fields [24]. In the context of radiopharmaceuticals, which contain radioactive isotopes used for diagnosing or treating diseases, integrating stimulus-responsive release systems can significantly enhance the precision, imaging capabilities, and therapeutic efficacy of these drugs while minimizing side effects and toxicity [25]. Various stimuli have been explored for the targeted delivery of radiopharmaceuticals. For instance, pH-sensitive nanocarriers, including liposomes, micelles, or polymers, can exploit the acidic environment of tumors to release radiopharmaceuticals selectively [26]. Temperature-sensitive materials, such as thermosensitive liposomes or polymers, respond to hyperthermia, improving nanocarrier permeability and activating the release of radiopharmaceuticals [27]. Redox-sensitive nanocarriers, like disulfide-linked micelles or polymers, release their payload in response to the higher levels of reactive oxygen species (ROS) in tumor cells. Light-sensitive nanocarriers, such as photo responsive liposomes or polymers, enable remote control over the release of radiopharmaceuticals by applying specific wavelengths, intensities, or durations of light. Additionally, magnetic-sensitive nanocarriers, like magneto liposomes or polymers, respond to external magnetic fields for targeted delivery and release [28]. The clinical significance of incorporating stimulus-responsive release systems in radiopharmaceuticals lies in their potential to enhance the accuracy and efficiency of nuclear medicine. These systems enable precise and controlled delivery of radioactive agents to the desired site and facilitate real-time monitoring and feedback of drug distribution and effects [29]. Potential applications include improved contrast and resolution in nuclear imaging modalities, enhanced therapeutic efficacy by delivering higher radiation doses to tumor cells while sparing normal tissues, and the integration of diagnosis and therapy in a theranostic approach. Recent research findings highlight the evolving landscape of stimulus-responsive release systems in radiopharmaceuticals. Examples include pH- and temperature-responsive liposomes for PET imaging and hyperthermia-induced drug release, redox- and light-responsive polymersomes for SPECT imaging and photodynamic therapy, and magnetic- and pH-responsive nanoparticles for MRI imaging and pH-triggered drug release [30]. These studies showcase the potential of such systems to improve tumor targeting, imaging, and therapeutic outcomes in preclinical models of breast cancer, melanoma, and glioma, respectively.

## Radiopharmaceuticals in imaging and theranostics

Radiopharmaceuticals have become a game-changer where diagnostics and treatment meet, pushing nuclear medicine into a time of incredible precision and patient-focused care. In the imaging world, these special drugs act like super-sensitive molecular probes, shining a light on the detailed picture of what's happening inside our bodies [31]. By smartly picking certain radioactive elements and teaming them up with molecules that matter in biology, these agents give us a sneak peek into the tiniest details of how our cells work. This helps catch diseases early, figure out their stage, and see how well treatments are working. The real magic happens when these drugs play a double role as theranostic agents, smoothly shifting from diagnosing to treating. Theranostics is like the guardian of personalized medicine, where these drugs are built to both find and fight diseases [32], as listed in Table 1. It is a tailored approach that aims to get the best treatment results while causing the least harm to healthy tissues. As we step into a new era in healthcare, radiopharmaceuticals in imaging and theranostics show us a perfect blend of scientific innovation, practical knowledge, and keeping patients at the centre. This is changing the way we do medicine, guiding us toward precise treatments that focus on effectiveness, safety, and the well-being of each person.

**Table 1:** Various applications of radiopharmaceuticals in imaging and theranostics

Application	Imaging purpose	Therapeutic purpose	References
Oncology	- Tumor detection and staging - Assessment of treatment response - Monitoring recurrence	- Targeted radiation therapy -Radionuclide therapy for metastatic lesions - Radioimmunotherapy for lymphomas	[36]
Cardiology	- Myocardial perfusion imaging - Evaluation of cardiac function	- Radio ablation of arrhythmias	[37]
Neurology	- Brain perfusion and metabolism imaging - Amyloid imaging in Alzheimer's disease - Neuroreceptor imaging for neurotransmitters	- Targeted radionuclide therapy for neuroendocrine tumors - Radiosynoviorthesis for inflammatory joint disorders - Pain palliation in bone metastases	[38]
Inflammation and infection imaging	- Infection localization - Assessment of inflammation	- Radioimmunotherapy for inflammatory diseases	[39]
Theranostics	- Personalized treatment planning - Real-time treatment monitoring	- Targeted radionuclide therapy in precision medicine - Image-guided therapies using theranostic agents	[40]
Bone imaging	- Bone metastases detection - Assessment of bone health and fractures	- Radiopharmaceuticals for bone pain relief	[41]

Healthcare professionals engaged in working with radiopharmaceuticals have access to specialized educational programs and certifications tailored to ensure their proficiency in the safe and effective handling of these substances. Numerous educational pathways are available for professionals seeking such expertise. One avenue includes Nuclear Medicine Technology Programs offered by universities and colleges, covering radiation safety, radio pharmacy, anatomy, physiology, and imaging techniques, often culminating in certifications from organizations like the American Registry of Radiologic Technologists (ARRT) or the Nuclear Medicine Technology Certification Board (NMTCB) [33]. Radio pharmacy Training programs focus on the preparation and handling of radiopharmaceuticals, and graduates may pursue certification as Authorized Nuclear Pharmacists (ANP) through the Board of Pharmacy Specialties (BPS). Radiation Safety Courses offer essential knowledge on principles, regulations, and practices, with certifications such as Certified Health Physicist (CHP) available through organizations like the American Board of Health Physics (ABHP) [34]. Continuing Education Programs keep professionals abreast of advancements, and organizations like the Society of Nuclear Medicine and Molecular Imaging (SNMMI) provide relevant courses. Advanced Degrees in Nuclear Medicine, including master’s or doctoral programs, offer in-depth knowledge and may contribute to career advancement. For those overseeing radiation safety, Radiation Safety Officer (RSO) Training programs and certifications, such as those from the American Academy of Health Physics (AAHP), equip individuals with the skills needed to manage safety protocols within healthcare facilities [35]. These diverse educational offerings collectively contribute to the development of a skilled workforce capable of ensuring patient care quality while prioritizing safety and compliance with regulations.

### Role of imaging in advancements

Radiopharmaceutical imaging plays a crucial role in advancing medicine, with diverse applications across various medical fields. In oncology, these specialized drugs help identify specific tumor markers, making it easier to locate and stage cancerous lesions [42]. This, in turn, enables the customization of targeted therapies and improves the monitoring of treatment effectiveness. In cardiology, radiopharmaceutical imaging is instrumental in assessing myocardial perfusion and function, leading to earlier detection of coronary artery disease, better risk evaluation, and enhanced patient outcomes [43]. The integration of radiopharmaceutical techniques with other diagnostic methods, such as the combination of PET and MRI, provides complementary anatomical and functional information, improving diagnostic accuracy in neurological disorders [44]. Beyond diagnosis, radiopharmaceutical imaging extends its influence into theranostics, where the same drug used for imaging also serves as a carrier for targeted radionuclide therapy. This emerging field holds promise for personalized treatment approaches tailored to patients' unique molecular profiles [45]. The intricate synergy between radiopharmaceutical development, imaging technology, and computational analysis has driven the creation of novel tracers and imaging protocols. As radiopharmaceutical chemistry evolves, producing compounds with enhanced specificity and longer half-lives, the potential for non-invasive imaging and therapy continues to grow [46]. In research, radiopharmaceutical imaging opens new avenues for investigating fundamental biological processes, providing valuable insights into disease mechanisms and treatment strategies. Additionally, it contributes to understanding drug pharmacokinetics and pharmacodynamics, facilitating drug development and optimization.

### Theranostics: healing through imaging-guided therapies

This innovative strategy harnesses the unique properties of radiopharmaceuticals, allowing for the simultaneous visualization and treatment of diseases at the molecular level. By utilizing radiotracers labelled with radioisotopes, clinicians can accurately identify specific biomarkers and assess the extent of disease progression through high-resolution imaging modalities such as PET and SPECT [47]. However, what sets radiopharmaceuticals theranostics apart is its transformative potential to go beyond diagnosis. The same radiotracers employed for imaging can be ingeniously tailored to deliver therapeutic payloads directly to diseased cells. This image-guided therapeutic approach ensures pinpoint accuracy in targeting malignant tissues while minimizing collateral damage to healthy organs. Radiopharmaceutical therapy, often termed “radionuclide therapy,” capitalizes on the precise localization of radiotracers, emitting therapeutic radiation that selectively destroys or halts the growth of cancerous cells [48]. This novel approach holds immense promise for refractory cancers and metastatic disease, where conventional treatments have shown limitations. The synergy between diagnostics and therapy in radiopharmaceuticals theranostics brings forth a new era of individualized medicine. Treatment regimens can be customized based on real-time imaging data, allowing clinicians to adapt and optimize therapies according to the patient’s response. This approach not only enhances treatment efficacy but also mitigates potential adverse effects, leading to improved patient outcomes and quality of life. In addition to oncology, radiopharmaceuticals theranostics demonstrate its versatility across various medical domains. In cardiology, it enables the assessment of myocardial perfusion and viability, aiding in the management of coronary artery disease [49]. Neurological applications encompass the diagnosis and monitoring of neurodegenerative disorders, with radiopharmaceuticals providing insights into neural function and degeneration patterns [50]. Beyond disease-oriented applications, radiopharmaceuticals theranostics plays a pivotal role in advancing our understanding of fundamental biological processes, offering a dynamic platform for pharmaceutical research and drug development. As we navigate the frontiers of modern medicine, radiopharmaceuticals theranostics emerges as a beacon of hope, illuminating the path toward more effective, personalized, and targeted therapeutic interventions. Its seamless integration of imaging and therapy, underpinned by precise radiotracer design and innovative instrumentation, holds the potential to reshape the landscape of medical practice, ultimately culminating in improved patient care and unprecedented medical breakthroughs [51].

## Safety and minimizing side effects

Ensuring the safety of those working with radiopharmaceuticals involves key considerations and strategies. Rigorous quality control measures are paramount, covering materials, personnel, and continuous result review to uphold the integrity of radiopharmaceutical preparation. Risk management guidelines, particularly from organizations like the European Association of Nuclear Medicine (EANM), provide insights into effectively handling risks associated with the small-scale preparation of radiopharmaceuticals [52]. Additionally, regulatory compliance is crucial, as the preparation and utilization of radiopharmaceuticals are subject to directives, regulations, and rules adopted by member states. In mitigating side effects, patient-specific dosing plays a pivotal role. Calculating the radiopharmaceutical dose based on the patient's condition is essential to minimize potential adverse reactions. Furthermore, close monitoring of patients during and after administration is vital for the prompt identification and management of any side effects. Guidelines and best practices further enhance safety in working with radiopharmaceuticals. Comprehensive training for all radiopharmacy staff, covering practices, preparation, quality control, and analytical techniques, is essential. The Society of Nuclear Medicine and Molecular Imaging (SNMMI) offers procedure guidelines, aiding nuclear medicine practitioners in establishing policies and procedures for radiopharmaceutical use [53]. The European Association of Nuclear Medicine (EANM) provides a guideline on Good Radiopharmacy Practice (cGRPP) for the small-scale preparation of radiopharmaceuticals. As radiopharmaceutical technologies continue to evolve and therapeutic paradigms expand, this concerted effort ensures that the benefits of radiopharmaceutical applications are harnessed to their fullest potential while prioritizing patient well-being and safety [54].

### Overcoming challenges in drug delivery

Radiopharmaceuticals represent a remarkable frontier in drug delivery, offering unique opportunities to address longstanding challenges in this field. The precision and specificity inherent in radiopharmaceuticals hold the potential to overcome hurdles that have hampered conventional drug delivery approaches. One of the key challenges in drug delivery has been achieving targeted delivery to specific tissues or cells while minimizing off-target effects. Radiopharmaceuticals, through their ability to selectively accumulate at disease sites based on molecular interactions, promise to revolutionize this aspect [55]. By engineering radiotracers with ligands that bind to disease-specific biomarkers, radiopharmaceuticals enable precise targeting, thus minimizing exposure of healthy tissues to therapeutic agents. Moreover, the dynamic imaging capabilities of radiopharmaceuticals offer real-time insights into drug distribution, pharmacokinetics, and therapeutic response. This imaging-guided approach facilitates dose optimization and the timely adjustment of treatment strategies, thereby overcoming issues of drug underutilization or overdose. Additionally, radiopharmaceuticals' versatility extends to crossing biological barriers that have previously hindered effective drug delivery [56]. Their small size, combined with diverse chemical structures, allows them to traverse the blood–brain barrier and other physiological barriers, enabling the delivery of therapeutic payloads to previously inaccessible sites. Radiopharmaceuticals also hold promise in addressing challenges related to drug resistance [57]. By delivering localized radiation directly to the target cells, they can circumvent mechanisms that confer resistance to traditional chemotherapies. Furthermore, the combination of diagnostics and therapy, as exemplified by theranostics, enables rapid assessment of treatment efficacy, guiding modifications to the therapeutic regimen if necessary.

### Minimizing off-target effects through precision

The intrinsic precision of radiopharmaceuticals, harnessed through meticulous design and strategic targeting, holds the potential to revolutionize therapeutic interventions. By conjugating therapeutic agents with radiotracers tailored to selectively bind to disease-specific molecular markers, radiopharmaceuticals confer an unprecedented degree of accuracy in drug delivery [58]. This strategic synergy not only enhances the therapeutic payload's localization to the intended site but also mitigates the risk of unintended interactions with healthy tissues, thus minimizing off-target effects that have plagued conventional treatments. Radiopharmaceuticals, guided by their inherent molecular specificity, navigate the intricate biological landscape with unparalleled discernment [59]. As they traverse physiological barriers and circulate throughout the body, radiotracers exploit the unique signaling pathways and surface receptors characteristic of disease cells, enabling precise homing to pathological foci. This precision is further accentuated by the real-time imaging capabilities of radiopharmaceuticals, facilitating dynamic visualization and quantification of their distribution patterns. Consequently, any divergence from the intended target can be promptly identified, permitting swift adjustments to treatment strategies to optimize therapeutic efficacy and curtail off-target effects. The rise of theranostics, a smart combination of diagnostics and therapy, showcases the highest level of precision made possible by radiopharmaceuticals [60]. This paradigm-shifting approach not only empowers clinicians to visualize disease at a molecular level but also empowers them to deliver therapeutics with pinpoint accuracy. As the field of radiopharmaceuticals continues to flourish, with advancements in radionuclide production, radiotracer design, and imaging modalities, the journey towards minimizing off-target effects gains remarkable momentum [61]. Collaborative endeavors across disciplines—from radiopharmacology and chemistry to medical physics and clinical practice—amplify the potential of radiopharmaceutical precision, underscoring their pivotal role in shaping the future of targeted therapies and redefining the concept of personalized medicine.

## Next-generation radiopharmaceutical formulations

The emergence of advanced radiopharmaceutical formulations signals a groundbreaking era in nuclear medicine, characterized by innovative progress in molecular design and delivery approaches [62]. These formulations, meticulously crafted through the intersection of radiopharmaceutical chemistry and state-of-the-art nanotechnology, have the potential to redefine diagnostic accuracy and therapeutic effectiveness. Using nanocarriers like liposomes, nanoparticles, and micelles, radiopharmaceuticals can be protected, ensuring their delicate contents remain intact, while also offering improved compatibility and extended circulation within the body [63]. Combining these carriers with disease-targeting ligands creates a unique balance of precision and adaptability, allowing radiotracers to uniformly navigate biological systems, providing unprecedented insights into cellular processes [64]. Moreover, these formulations, often designed for multifunctionality, allow for the simultaneous delivery of therapeutic agents, contrast agents, or even multiple radionuclides, showcasing remarkable flexibility and synergistic therapeutic potential [65].

Next-generation radiopharmaceuticals go beyond overcoming physiological barriers by being engineered to exploit endocytosis or receptor-mediated uptake pathways, granting access to cells and tissues that were once challenging to reach. The association of radiopharmaceuticals with emerging imaging modalities, such as theranostic applications utilizing PET/MR or SPECT/CT, combines anatomical and functional information, enhancing diagnostic accuracy and guiding personalized treatment strategies. Additionally, the introduction of radiolabelling techniques utilizing bioorthogonal chemistry, click chemistry, and prosthetic groups for site-specific attachment further enhances the precision and stability of radiopharmaceuticals [66]. As these next-gen radiopharmaceutical formulations take centre stage in medical innovation, interdisciplinary collaborations between chemists, radiopharmacologists, nanotechnologists, and clinicians drive the evolution of these remarkable agents [67]. The future looks promising as we witness the fusion of molecular innovations and technological prowess, ushering in a new era where the therapeutic and diagnostic capabilities of radiopharmaceuticals are expanded beyond imagination. Table 2 lists the application of radiopharmaceuticals in drug delivery.

**Table 2:** Role of radiopharmaceuticals in drug delivery

Application	Radiopharmaceutical role	Description	References
Cancer therapy	Radiolabelling of nanoparticles for targeted imaging and therapy	- Targeted delivery of chemotherapeutic agents to tumors - Enhanced permeability and retention effect utilization - Personalized treatment with controlled drug release - Combination therapy for synergistic effects	[68]
Imaging-guided therapies	Incorporation of radiotracers for real-time monitoring	- Simultaneous imaging and therapy for real-time monitoring - Image-guided drug release using external triggers - Theranostic agents for precise diagnosis and treatment	[69]
Neurological disorders	Radiopharmaceutical-guided nanoparticle delivery	- Blood–brain barrier crossing for central nervous system drug delivery - Targeted therapy for neurodegenerative diseases	[70]
Inflammatory conditions	Radiolabelled nanoparticles for tracking and therapy	- Targeted delivery of anti-inflammatory agents - Nanoparticle-based immune modulation	[71]
Cardiovascular applications	Radiolabelling to assess nanoparticle distribution	- Drug delivery to diseased blood vessels - Reduction of systemic side effects	[72]
Gene delivery	Radiopharmaceutical labeling for tracking gene delivery	- Nucleic acid delivery for gene therapy - Targeted gene editing and modulation	[73]
Personalized medicine	Radiotracer-guided customization of drug delivery	- Tailored drug delivery based on patient-specific markers - Customized treatment regimens	[74]
Minimizing side effects	Radiolabelling for tracking nanoparticle biodistribution	- Localized drug release to reduce systemic toxicity - Controlled and sustained drug delivery	[75]
Combination therapies	Radiopharmaceutical tracking of combination therapy	- Simultaneous delivery of multiple therapeutic agents - Synergistic effects for enhanced efficacy	[76]
Regenerative medicine	Radiolabelling for monitoring tissue repair	- Controlled delivery of growth factors for tissue repair - Stem cell therapy augmentation with targeted drugs	[77]

### Nanoparticle artistry in drug delivery

**Fig. 2:**
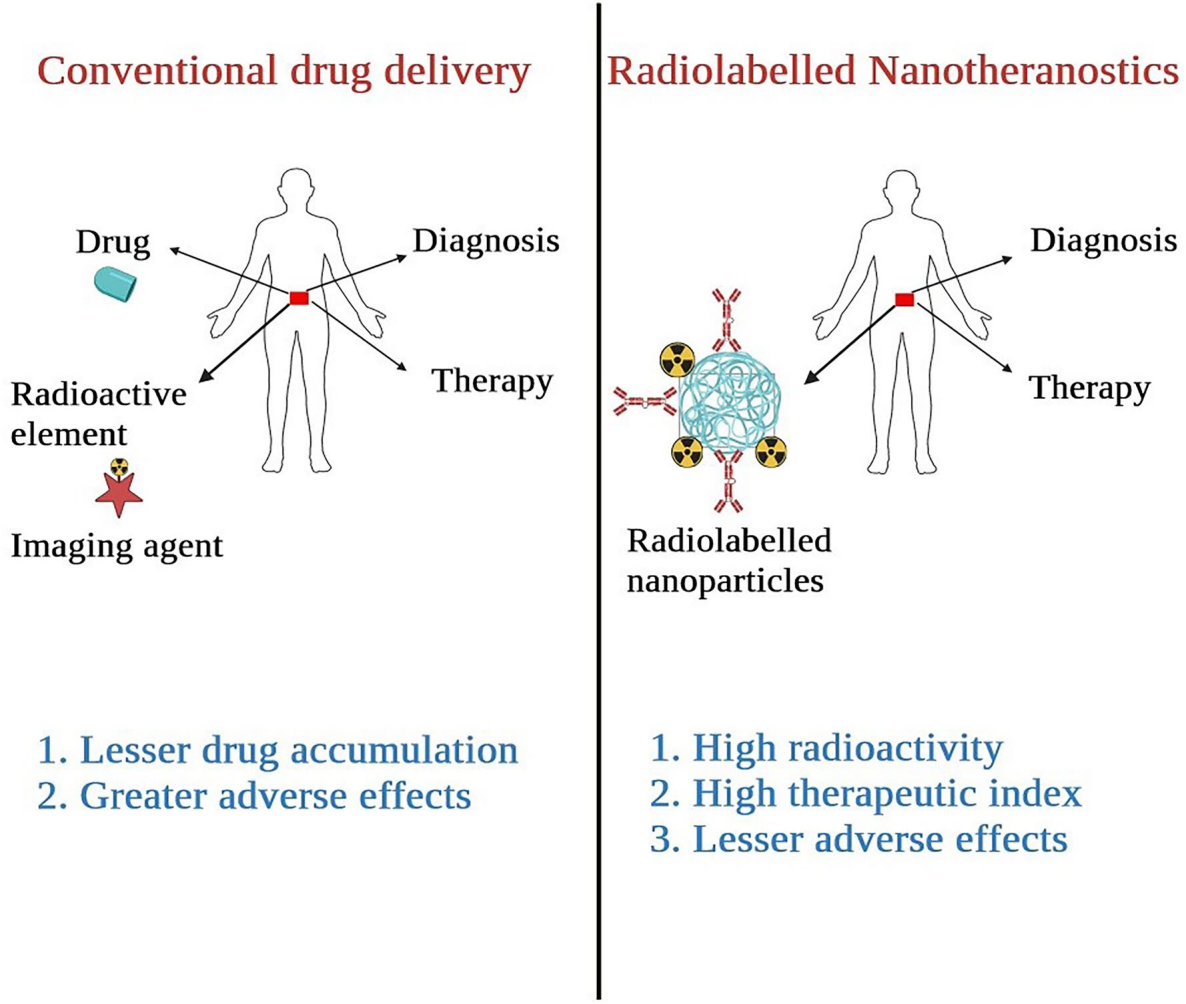
Current strategies and Radiopharmaceuticals application in theranostics

Radiopharmaceuticals, driven by the science of nanoparticle engineering, are on the brink of transforming drug delivery with unprecedented precision. The union of radiopharmaceutical science and nanotechnology gives rise to a wave of innovation, where precisely designed nanoparticles act as sophisticated carriers, orchestrating the targeted delivery of therapeutic and diagnostic payloads [78]. Engineered with tailored surface modifications and ligands for disease-specific recognition, these nanoparticles navigate biological landscapes with remarkable accuracy, evading clearance mechanisms and homing in on unique cellular receptors or microenvironments in pathological tissues [79]. This fusion not only improves therapeutic efficacy by concentrating agents at disease sites but also minimizes off-target effects, a challenge in conventional drug delivery. The synergy of radiolabelling techniques with nanoparticle formulations amplifies their diagnostic potential, as radionuclide-bearing nanoparticles illuminate molecular processes in vivo with unparalleled resolution [80]. Nanoparticle radiopharmaceuticals go beyond being carriers, revealing multifunctionality that enhances their capabilities. These platforms allow the co-delivery of therapeutic agents, contrast agents, or multiple radiotracers, creating finely tuned symphonies of treatment and imaging tailored to individual patient profiles [81]. The modularity of nanoparticle design, combined with their inherent physicochemical properties, enables radiopharmaceuticals to cross biological barriers that traditionally hindered drug delivery [82]. However, despite their promise, challenges, and limitations persist in using nanoparticles as delivery vehicles for radiopharmaceuticals. Issues such as potential toxicity, stability, and scalability of production need careful consideration. Achieving consistent and reproducible results, as well as addressing concerns related to long-term safety, remain areas of active research and development in this evolving field. As radiopharmaceutical nanoparticles advance, the intersection of art and science in their design becomes increasingly evident, depicting a canvas where therapeutic precision and diagnostic acuity harmoniously converge. Collaborations among radiopharmacologists, nanotechnologists, chemists, and clinicians play a pivotal role in propelling these innovations into uncharted territories of personalized medicine. Each stroke of innovation brings us closer to unraveling the mysteries of disease and the art of healing. Figure 2 depicts the difference between the conventional and radiolabelled treatment strategies.

Nanoparticles-based radiopharmaceuticals delivery systems have emerged as a transformative approach in the field of nuclear medicine. These systems, including tiny particles like organic and inorganic nanoparticles, monoclonal antibodies, and microspheres, use the special features of nanoparticles—like their size and surface—to make radiopharmaceuticals work better [83]. For example, nanoparticles can make drugs more available, less toxic, and more efficient. They can also be customized with different molecules for precise targeting [84]. Because they have a larger surface area, nanoparticles can carry more radiopharmaceuticals, making treatments more effective. Recent studies highlight the potential of these systems in treating diseases, especially in cancer diagnosis and management [85]. But there are challenges in making these systems work perfectly, like improving how well they target specific areas, creating strong methods for including radionuclides, ensuring they're safe, and dealing with regulations. Despite these challenges, the use of nanoparticle-based delivery for radiopharmaceuticals is growing fast, opening up exciting possibilities in nuclear medicine.

## Radiopharmaceutical case studies

Radiopharmaceuticals, radioactive agents designed to target and treat diseases, particularly cancer, have shown significant clinical impact, as illustrated by several case studies: One notable example is the use of ^177^Lu-PSMA-617 in metastatic castration-resistant prostate cancer (mCRPC). This PSMA-targeted radiopharmaceutical delivers beta radiation to prostate cancer cells. In the phase III VISION trial, ^177^Lu-PSMA-617, when combined with standard care, demonstrated a substantial improvement in overall survival and radiographic progression-free survival compared to standard care alone in patients with PSMA-positive mCRPC. The median overall survival increased from 11.3 to 15.3 months, and the median radiographic progression-free survival extended from 3.4 to 8.7 months [86]. Common adverse events included hematologic, gastrointestinal issues, and fatigue.

Another impactful case involves 223Ra-dichloride used for bone metastases from prostate cancer. This alpha-emitting radiopharmaceutical, mimicking calcium, accumulates in areas of increased bone turnover, such as bone metastases. In the phase III ALSYMPCA trial, 223Ra-dichloride demonstrated improved overall survival and delayed symptomatic skeletal events in patients with castration-resistant prostate cancer and bone metastases. Median overall survival increased from 11.3 to 14.9 months, and the median time to the first symptomatic skeletal event extended from 9.8 to 15.6 months [87]. Common adverse events included nausea, diarrhea, vomiting, and hematologic issues.

In the case of neuroblastoma, ^131^I-metaiodobenzylguanidine (^131^I-MIBG), a radiopharmaceutical targeting the norepinephrine transporter highly expressed in neuroblastoma cells, showed promise in a phase II trial. When combined with chemotherapy and stem cell rescue, ^131^I-MIBG improved event-free survival and overall survival in children with high-risk neuroblastoma. The 3-year event-free survival increased from 48 to 61%, and the 3-year overall survival improved from 59 to 74% [88]. Common adverse events included hematologic issues, infections, and mucositis. These case studies underscore the potential of radiopharmaceuticals to enhance outcomes and improve the quality of life for patients across various diseases.

### Radiolabelled antibodies targeting solid tumors

**Fig. 3:**
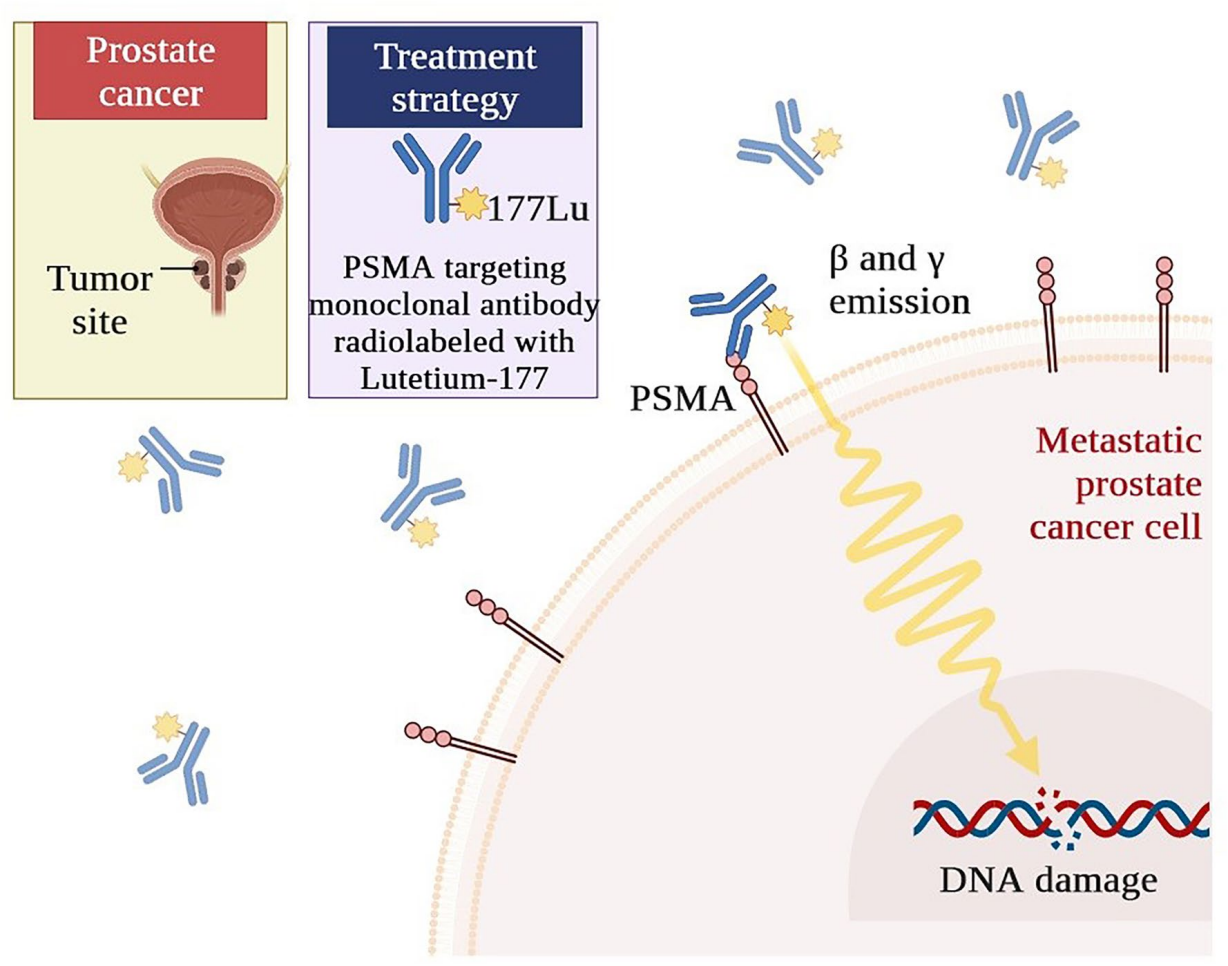
Radioligand therapy for metastatic prostate cancer treatment

Radiopharmaceuticals highlight significant progress in targeted cancer therapy, utilizing radiolabelled antibodies for solid tumors [89]. These stories emphasize the potential of combining monoclonal antibodies’ specificity with radiopharmaceutical precision for tailored and effective treatments, particularly in cases of refractory or metastatic solid tumors [90]. An excellent example is ^177^Lu-DOTATATE, a radiolabelled somatostatin analogue, used to treat neuroendocrine tumors (NETs) [91]. This approach capitalizes on somatostatin receptors’ overexpression on NET cells, delivering targeted radiation to tumor sites while minimizing damage to healthy tissues [92]. Radiopharmaceutical case studies demonstrate the efficacy of ^177^Lu-DOTATATE in halting tumor progression and inducing regression, providing evidence for its inclusion in therapeutic protocols [93]. Additionally, radiolabelled antibodies like ^131^I-rituximab have paved the way for innovative treatment strategies for non-Hodgkin lymphomas, showcasing the power of radioimmunotherapy [94]. Combining a monoclonal antibody with a therapeutic radionuclide allows targeted destruction of lymphoma cells, enhancing treatment outcomes and minimizing systemic toxicity [95]. Case studies illustrate instances where radioimmunotherapy achieved notable remission rates, offering hope for patients with limited therapeutic options. Consider the compelling example of HER2-targeted radioimmunotherapy for HER2-positive breast cancer. Conjugating radionuclides to trastuzumab, a monoclonal antibody specific to HER2 receptors, has demonstrated the potential to deliver localized radiation directly to tumor cells, enhancing treatment efficacy and reducing systemic side effects [96]. Radiopharmaceutical case studies vividly illustrate instances where this approach resulted in tumor regression and prolonged survival, underscoring the promise of such tailored strategies [97]. In prostate cancer, radiolabelled antibodies like ^177^Lu-PSMA-617 have emerged as potent tools, capitalizing on the overexpression of prostate-specific membrane antigen (PSMA) in prostate cancer cells [98], as depicted in Fig. 3. These radiopharmaceuticals have shown impressive results in targeted radionuclide therapy, with metastatic castration-resistant prostate cancer patients experiencing significant declines in prostate-specific antigen (PSA) levels and improved quality of life. The elegance of radiolabelled antibodies lies not only in their targeting capabilities but also in their potential to be combined with other therapeutic modalities, amplifying their impact [99]. These radiopharmaceuticals stand as powerful examples of the potential transformation achieved through the combination of radiolabelled antibodies and solid tumors [100]. As these narratives unfold, they highlight the harmonious blend of scientific innovation and compassionate patient care, providing a glimpse into the future of oncology where radiopharmaceuticals are set to reshape treatment approaches and redefine the precision medicine landscape.

### PRRT: neuroendocrine tumor treatment

Radiopharmaceutical case studies shed light on the significant clinical advancements brought about by Peptide Receptor Radionuclide Therapy (PRRT) in treating neuroendocrine tumors (NETs), marking a paradigm shift in oncology. PRRT leverages radiolabelled somatostatin analogues like ^177^Lu-DOTATATE, utilizing their molecular specificity to target tumor cells overexpressing somatostatin receptors [101]. These cases highlight instances where PRRT achieved remarkable therapeutic responses, demonstrating its efficacy by delivering targeted radiation to NET cells while sparing healthy tissues [102]. The studies depict scenarios of substantial tumor regression, symptom relief, and extended progression-free survival in advanced NET patients. PRRT’s theranostic capabilities, where the same radiotracer used for diagnosis guides therapy, underscore the precision of this approach [103]. These case studies underscore PRRT's transformative potential, showcasing the synergy of advanced radiopharmaceutical science and compassionate patient care, paving the way for a future where PRRT plays a crucial role in managing neuroendocrine tumors. The depicted scenarios reveal significant improvements in symptoms such as hormonal hypersecretion or pain, translating into meaningful enhancements in patients’ well-being. PRRT’s ability to interact with diverse somatostatin receptor subtypes adds complexity, demonstrating its versatility in addressing various NET manifestations [104]. Moreover, the interplay between PRRT and other therapeutic modalities, such as surgery or targeted therapies, emerges as a recurring theme. The cases illustrate instances where PRRT acts synergistically, either as neoadjuvant therapy before surgery or alongside other targeted agents to enhance therapeutic outcomes [105]. As radiopharmaceutical science advances and our understanding of tumor biology deepens, PRRT’s potential continue to grow. Collaborations between nuclear medicine specialists, radio pharmacists, and oncologists fuel these case studies, highlighting PRRT's role in shaping a new era of precision oncology.

### Managing metastases in bones

Radiopharmaceutical case studies reveal a groundbreaking frontier in addressing bone metastases, showcasing the profound impact of precision medicine in oncology. These studies highlight the crucial role of radiopharmaceuticals in diagnosing, staging, and providing targeted therapies for patients dealing with bone metastases. Notably, cases using bone-seeking radiotracers like technetium-99 m bisphosphonates demonstrate how these agents effectively outline the extent and distribution of metastatic lesions, aiding in accurate disease characterization and treatment planning [106]. Striking examples emerge where targeted radiopharmaceutical therapies, such as strontium-89 chloride or samarium-153 lexidronam, deliver focused radiation to osteoblastic or osteolytic lesions, offering relief from pain and promoting skeletal stability [107]. Radiopharmaceutical case studies intricately showcase multimodal approaches, illustrating how radiopharmaceuticals seamlessly integrate with other therapeutic modalities [108]. Cases demonstrate the synergistic effects of combining radiopharmaceutical treatments with systemic therapies, surgical interventions, or external beam radiation, expanding the spectrum of therapeutic options for patients with bone metastases. Furthermore, theranostic paradigms, exemplified by radiolabelled prostate-specific membrane antigen (PSMA) ligands in prostate cancer, epitomize the exquisite precision of radiopharmaceuticals [109]. These dual-action agents enable accurate staging and localization of metastatic foci, facilitating tailored therapies that leverage radiopharmaceuticals for both diagnostic insight and targeted therapeutic intervention. Cases utilizing radiolabelled bisphosphonates, like ^99mTc-MDP, highlight their pivotal role in guiding therapeutic decisions by providing an accurate assessment of tumor burden and bone turnover [110]. In instances where bone-seeking radiopharmaceuticals are used for palliative care, they showcase how selective irradiation of metastatic lesions, tailored to the patient's individual needs, leads to remarkable pain relief and improved quality of life. The dynamic landscape of bone-targeted radiopharmaceuticals is further emphasized through cases involving alpha-emitting radionuclides, like ^223Ra, which leverage the unique properties of these particles to selectively irradiate bone metastases while minimizing damage to surrounding tissues [111]. These case studies underscore the potential of alpha-emitting radiopharmaceuticals to induce tumor regression and delay disease progression, offering a glimpse into the therapeutic horizons they unveil. These case studies serve as testimonials to the profound impact of radiopharmaceuticals in navigating the complex landscape of bone metastases.

## Seminal advances and pioneering developments in the field of radiopharmaceuticals

Radiopharmaceuticals, consisting of radioisotopes bound to biological molecules, exhibit a targeted approach to specific organs, tissues, or cells within the human body, serving dual roles in disease diagnosis and therapy. In recent years, notable strides have been made in the realm of radiopharmaceuticals, with significant ongoing work in key areas. Firstly, the production of over 100 radiopharmaceuticals has been a focal point, involving the use of radioisotopes from nuclear research reactors or cyclotrons [112]. The International Atomic Energy Agency (IAEA) plays a crucial role in assisting Member States in enhancing their capacities for localized radiopharmaceutical production. Secondly, there is a burgeoning focus on radiopharmaceuticals for cancer therapy, constituting a novel drug class delivering radiation therapy directly to cancer cells. Research and clinical trials in this domain have surged, indicating the potential to minimize side effects and target minute cancer cell deposits throughout the body [113]. Lastly, diagnostic radiopharmaceuticals have gained prominence, offering valuable insights into organ function and disease presence [114]. These examples encapsulate the pioneering work unfolding in the radiopharmaceutical field, presenting exciting prospects for the progress of nuclear medicine as research and development continue to expand these horizons.

As an author we view the ongoing works and advancements with a sense of enthusiasm and optimism. The production of a diverse range of radiopharmaceuticals, facilitated by the collaboration with organizations like the IAEA, is a testament to the global efforts to meet the local demands for these crucial medical agents. The progress in developing radiopharmaceuticals for cancer therapy is particularly promising, heralding a new era in precision medicine where targeted radiation therapy could potentially revolutionize cancer treatment, minimizing side effects and enhancing therapeutic efficacy. The increasing use of radiopharmaceuticals for diagnostic purposes adds another layer of significance to these developments. The ability to gather valuable information about organ function and disease presence contributes significantly to early and accurate disease detection, paving the way for timely interventions. These advancements collectively represent a transformative shift in the landscape of nuclear medicine. Looking ahead, the continuous expansion of research and clinical trials in radiopharmaceuticals inspires a sense of anticipation. The potential for reducing the risks associated with conventional radiation therapy, as well as the exploration of new avenues for cancer treatment and disease diagnosis, underscores the dynamic and innovative nature of the field. We believe that as these works progress, the field of radiopharmaceuticals will not only address current healthcare challenges but also open doors to novel and personalized approaches in disease management.

## Future of radiopharmaceuticals

The future of radiopharmaceuticals is an exciting story of scientific innovation set to transform medical practices. This journey spans precision medicine, molecular imaging, and targeted therapies, converging to reveal once unprecedented advancements. Radiopharmaceuticals, driven by collaborations between radio pharmacists, nuclear medicine specialists, chemists, and bioengineers, are on the brink of expanding their horizons [115]. With clever radiotracer design and engineering, these agents promise a leap in diagnostic accuracy, enabling real-time visualization of cellular processes at the molecular level. Artificial intelligence and machine learning further enhance radiopharmaceuticals’ potential for predictive modelling, image analysis, and treatment optimization. The emergence of novel radionuclides with improved properties amplifies the diagnostic precision and therapeutic efficacy of radiopharmaceuticals [116]. Moreover, the evolution of personalized radiopharmaceutical therapies, like theranostics, marks an era where each patient's unique molecular profile guides tailored treatment regimens for improved outcomes.

**Fig. 4:**
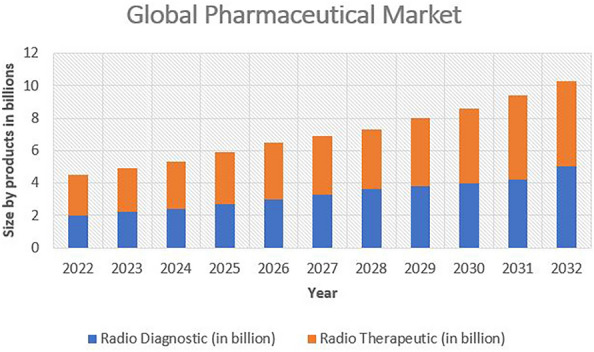
Forecast of the global pharmaceutical market from the year 2022–2032

The future of radiopharmaceuticals goes beyond disease diagnosis and treatment, expanding into drug development and basic research [117]. Radiopharmaceuticals provide a transformative lens to explore intricate biological pathways, study drug pharmacokinetics, and assess treatment responses in preclinical models, streamlining the drug discovery process [118]. Integrating radiopharmaceutical technologies with nanotechnology, gene therapy, and immunotherapy opens new frontiers in combating diseases that have long evaded conventional interventions [119]. As the future unfolds, radiopharmaceuticals would take centre stage in the transition from generalized medicine to tailored therapies. The collaborative blend of molecular precision, advanced imaging technologies, and multidisciplinary collaborations propels radiopharmaceuticals to the forefront of medical innovation [120]. These agents, driven by scientific insight and a commitment to patient well-being, reveal a future where the boundaries of possibility are pushed beyond imagination, revolutionizing the essence of healthcare. Figure 4 highlights the future of radiopharmaceuticals in diagnosis and as a therapeutic agent.

### Precision through artificial intelligence

The partnership of radiopharmaceuticals and AI marks a powerful collaboration, merging the intricacies of molecular targeting with the analytical strength of machine learning algorithms. This partnership boosts the accuracy of radiopharmaceutical imaging, with AI algorithms efficiently navigating through vast datasets to reveal subtle patterns and anomalies that might escape human detection [121]. The combination of AI-driven image analysis and radiopharmaceuticals signifies a new era, characterized by improved lesion detection, precise disease staging, and early evaluation of treatment effectiveness. AI’s role extends to optimizing the planning of radiopharmaceutical therapy, incorporating patient-specific factors, radiotracer biodistribution profiles, and dosimetry calculations [122]. This results in tailored treatments of unparalleled accuracy and efficacy. The fusion of AI with radiopharmaceuticals not only enhances diagnostic and therapeutic outcomes but also accelerates research and drug development, transforming the discovery of novel radiotracers and therapeutic agents [123]. As AI continues to evolve, its collaboration with radiopharmaceuticals paints a picture of a future where personalized medicine becomes a tangible reality. This partnership reshapes the landscape of patient care, combining the precision of molecular science with the limitless capabilities of technology. The journey ahead holds the promise of a healthcare paradigm where AI-guided radiopharmaceuticals light the way to optimal diagnosis, treatment, and, ultimately, improved patient well-being.

### Future directions and emerging trends

Radiopharmaceuticals, incorporating radioactive isotopes, have emerged as promising tools in the medical realm, particularly for the diagnosis and treatment of diseases like cancer. However, as we delve into the future of these agents, certain directions and trends come to the forefront. Firstly, the improvement of supply chain management stands as a critical focus. Ensuring the efficient production and distribution of radiopharmaceuticals, particularly those with short half-lives, is paramount for accessibility [124]. Strategies involving redundancy planning, upscaling, and infrastructure development are being explored to address this challenge. Another avenue of exploration involves the quest for new radioisotopes and radiopharmaceuticals. Researchers are actively investigating alternatives such as alpha emitters, theranostics, and multimodal agents, offering intriguing opportunities for both imaging and therapy while potentially overcoming existing limitations. In parallel, there is a dedicated effort towards advancing dosimetry and radiobiology. The development and standardization of methods and parameters for measuring and optimizing radiation dose and biological effects are crucial for ensuring the safety and efficacy of radiopharmaceuticals [125]. This includes addressing challenges related to normal organ dose limits, individualized dosimetry, and radiobiological models. Additionally, the regulatory and licensing landscape surrounding radiopharmaceuticals is undergoing scrutiny. Harmonizing and streamlining these processes, especially for innovative products, is essential for their smooth clinical translation and market access. This necessitates enhanced collaboration among developers, scientists, regulators, manufacturers, and private enterprises to navigate these regulatory and licensing intricacies. As we chart the future of radiopharmaceuticals, these directions underscore the commitment to advancing their clinical utility and overcoming challenges in the ever-evolving landscape of medical science.

### Economic considerations

The cost-effectiveness of using radiopharmaceuticals in healthcare is a multifaceted consideration that involves weighing both the economic benefits and potential challenges. While radiopharmaceuticals play a pivotal role in disease diagnosis, treatment, and therapeutic monitoring, their production and implementation involve intricate processes that can contribute to higher upfront costs [126]. However, the long-term economic benefits often outweigh these initial expenditures. Radiopharmaceuticals, particularly in the context of nuclear medicine procedures, can provide precise and early diagnostic information, leading to more targeted and effective treatment strategies. This, in turn, may result in reduced overall healthcare costs by minimizing unnecessary interventions and enhancing treatment success rates [127]. Additionally, the emergence of theranostics, which combines diagnostics and therapy using radiopharmaceuticals, has the potential to further optimize treatment regimens, potentially reducing the economic burden associated with prolonged or ineffective therapies [128]. Striking a balance between upfront costs and long-term benefits is crucial, and ongoing advancements in radiopharmaceutical research and technology may contribute to enhancing their overall cost-effectiveness in healthcare applications.

## Conclusions

Radiopharmaceuticals represent a remarkable fusion of science and innovation, ushering in a transformative era in medicine. The evolution of radiolabelling techniques has allowed for the precise development of radiopharmaceuticals, paving the way for targeted drug delivery. Strategies like passive targeting, active targeting, and stimulus-responsive release systems have redefined therapeutic precision, emphasizing safety and minimizing off-target effects. The integration of nanoparticle engineering adds an artistic touch to drug formulations, promising a revolution in drug delivery. Radiopharmaceutical case studies spanning diverse therapeutic domains showcase significant progress in cancer treatment and beyond. Looking ahead, the fusion of radioisotopes and pharmaceuticals holds the promise of catalysing a new era of targeted therapeutics. Advances in nanotechnology and biomolecular engineering, coupled with the strategic interplay of passive and active targeting, provide unprecedented accuracy in engaging diseases at their molecular epicentre. Stimulus-responsive release systems offer fine-tuned drug administration tailored to individual patient microenvironments. In the realm of imaging and theranostics, radiopharmaceuticals are set to wield enhanced diagnostic and therapeutic potential, optimizing patient outcomes. The integration of radiopharmaceuticals with AI-driven technologies promises a healthcare future where individualized medicine and groundbreaking discoveries become the norm. In this symphony of scientific evolution, radiopharmaceuticals stand as a harmonious melody, shaping a future that transcends boundaries, optimizes outcomes, and redefines the trajectory of medical progress.

## Data Availability

Not applicable.
